# Controlling COVID-19 Outbreaks with Financial Incentives

**DOI:** 10.3390/ijerph18020724

**Published:** 2021-01-15

**Authors:** Chaeyoung Lee, Soobin Kwak, Junseok Kim

**Affiliations:** Department of Mathematics, Korea University, Seoul 02841, Korea; chae1228@korea.ac.kr (C.L.); soobin23@korea.ac.kr (S.K.)

**Keywords:** SUC epidemic model, COVID-19, financial incentives, least-squares fitting

## Abstract

In this paper, we consider controlling coronavirus disease 2019 (COVID-19) outbreaks with financial incentives. We use the recently developed susceptible-unidentified infected-confirmed (SUC) epidemic model. The unidentified infected population is defined as the infected people who are not yet identified and isolated and can spread the disease to susceptible individuals. It is important to quickly identify and isolate infected people among the unidentified infected population to prevent the infectious disease from spreading. Considering financial incentives as a strategy to control the spread of disease, we predict the effect of the strategy through a mathematical model. Although incentive costs are required, the duration of the disease can be shortened. First, we estimate the unidentified infected cases of COVID-19 in South Korea using the SUC model, and compute two parameters such as the disease transmission rate and the inverse of the average time for confirming infected individuals. We assume that when financial incentives are provided, there are changes in the proportion of confirmed patients out of unidentified infected people in the SUC model. We evaluate the numbers of confirmed and unidentified infected cases with respect to one parameter while fixing the other estimated parameters. We investigate the effect of the incentives on the termination time of the spread of the disease. The larger the incentive budget is, the faster the epidemic will end. Therefore, financial incentives can have the advantage of reducing the total cost required to prevent the spread of the disease, treat confirmed patients, and recover overall economic losses.

## 1. Introduction

**Figure 1:**
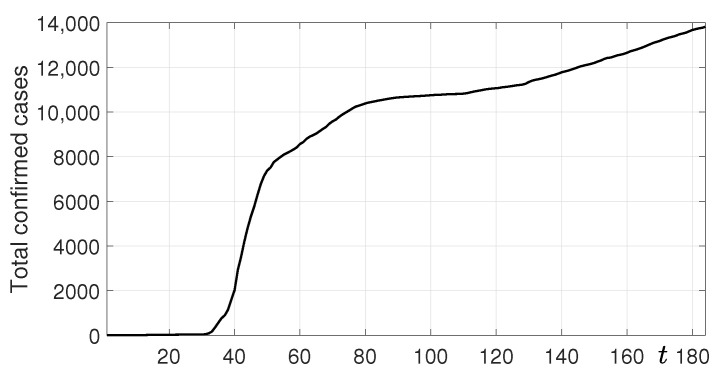
Total confirmed cases of COVID-19 in South Korea from 20 January to 17 July 2020.

The coronavirus disease 2019 (COVID-19), first reported in Wuhan, China, in December 2019, has quickly spread worldwide [1,2]. More than 13 million patients have been diagnosed and many people, nearly 600,000, have died as of 17 July 2020 [3]. In South Korea, since the report of the first patient on 20 January 2020, more than 13,000 patients have been confirmed as of 17 July 2020 (see Figure 1). The data were reported by the Korea Disease Control and Prevention Agency (KDCA) [4].

In the early stages of COVID-19 spread, confirmed patients in South Korea were mostly infected abroad. However, since then, the number of newly confirmed cases has rapidly increased through the spread in the community, leading to cluster cases. If one suspected case of COVID-19 occurs and the diagnosis is delayed, the infection spreads rapidly to the community through family, acquaintances, coworkers, and users of facilities shared by a large number of people; hence, the number of infected people increases sharply.

To prevent the spread of COVID-19, governments from different countries implemented national strategies [5,6]. Further, it is essential to use a face mask and wash hands to protect the respiratory organs and eyes [7], maintain physical distance between individuals to avoid contact with others as much as possible [8,9,10], and isolate the infected [11]. Right after suspected symptoms are observed, voluntary testing is significantly effective in averting secondary infection [12]. Asymptomatic infection, one of the possible cases of COVID-19, may have delayed the initial detection of the disease by preventing recognition of the infection being present [13,14]. In some cases, even though the symptoms of COVID-19 were recognized, the unidentified confirmed people were not self-isolated, continued to go to work, and visited public and multi-purpose facilities while hiding the symptoms, coming into contact with many susceptible people. As a result, many susceptible people came in contact with the virus and were infected.

In South Korea, if anyone who has been in close contact with the confirmed patients or if there is a doctor’s opinion that the infection is suspected, then it is a legal requirement to get tested, which is also valid in Australia and other countries. If those who comply with COVID-19 preventive regulations are confirmed positive after testing, then all the costs of examination, quarantine, and treatment are paid by the Korea National Health Insurance Service, the central government, and local governments. Therefore, the patients do not need to worry about the costs of COVID-19 diagnosis and treatment. Regarding the livelihood, in addition, those who are hospitalized or quarantined in connection with the outbreak of the confirmed cases of COVID-19 can receive paid leave from their employers or receive living support expenses from the government.

Nonetheless, some people refuse to take the COVID-19 diagnostic test, even if they have the main symptoms or know that they have possibly come into contact with confirmed patients. There are several reasons for this: some people are not really aware of the seriousness of the epidemic, others are afraid of being disadvantaged at work or losing their job due to long-term isolation after being confirmed, and others are apprehensive of being criticized by others when they are confirmed. Infected people with these thoughts can result in cluster cases and lead to difficulty in clearly ascertaining the paths of infection. According to the results of [15], individuals in the incubation period of the disease might spread the disease rapidly; such individuals have a significant impact on the spread of this infectious disease. To prevent this, therefore, individuals must improve their awareness of the risks of the disease. We can encourage people with suspected symptoms to take COVID-19 tests voluntarily by providing financial incentives to patients who test positive from voluntary tests. Of course, in order to avoid moral problems, the right standards for monetary compensation must also be prepared. For example, the compensation is only provided to the confirmed cases who know the route of infection. In fact, in some countries, the local governments have decided to provide minimum monetary awards to COVID-19 patients confirmed through voluntary testing. However, cases that were already confirmed were excluded from these compensations [16].

Financial incentives can be a catalyst to increase the voluntary testing of individuals and can play a vital role in limiting the rapid spread of the infection at a lower cost if monetary rewards are given. In [17], incentives were introduced, and types of effectiveness were studied to increase the retest rate of people classified as high-risk groups after the first examination. The study estimated that monetary incentives could be applied at the efficient cost of potentially controlling the spread of the disease and that, as a result of the simulation, patients who received incentives were less contagious [18]. The effects of financial incentives and disease compensation were analyzed in [19]; they suggested that economic incentives would promote vaccinations in farms and reduce the spread of the disease.

To prevent the spread of COVID-19, there is one more thing just as important as having people at risk of infection being tested voluntarily. This is to ensure that test results are rapid, both to minimize the disincentive of any pre-emptive isolation and also to ensure timely contact tracing. The timeliness of test results is also crucial, hence, we also consider that the financial incentives should be offered to laboratories, medical staff, or staff for epidemiological investigation, etc. Rapid results would have a similar effect on rewarding the confirmed cases in preventing the spread of COVID-19.

The main purpose of this study is to investigate how financial incentives affect the spread of the epidemic by using a mathematical epidemic model. We first estimate the unidentified infected cases of COVID-19 in South Korea using a recently developed epidemic model, the susceptible-unidentified infected-confirmed (SUC) model [20]. The unidentified infected are defined as the infected but not identified people who are not yet isolated and can transmit the disease to the susceptible. In addition to the SUC model, various mathematical models for infectious diseases have been studied [21,22,23,24,25,26]. Nevertheless, we use the SUC model to consider the unconfirmed infections for controlling the highly contagious COVID-19 more effectively. Therefore, we propose a modified SUC model to compute the impact of incentive policies in preventing the spread of the disease. It is assumed that more people who are infected but not identified will be rapidly tested as positive if the financial incentives are provided. We then examine the effect of increased testing on the spread of the infectious disease.

The rest of the paper is organized as follows. In Section 2, the proposed mathematical model is described, and its numerical solution algorithm is presented. In Section 3, the results of the numerical tests are presented. In Section 4, we discuss the numerical results and present the merits and limitations of the proposed mathematical model. In Section 5, we provide our conclusions. Note that the data used are listed in Appendix A and the MATLAB code for the numerical implementation is provided in Appendix B.

## 2. Mathematical Model and Numerical Solution Algorithm

The SUC epidemic model [20] is as follows:

(1) $$\begin{array}{ccc} \frac{dS(t)}{dt} & = & -\beta \frac{S(t)U(t)}{N}, \end{array}$$

(2) $$\begin{array}{ccc} \frac{dU(t)}{dt} & = & \beta \frac{S(t)U(t)}{N}-\gamma U\left(t\right), \end{array}$$

(3) $$\begin{array}{ccc} \frac{dC(t)}{dt} & = & \gamma U(t), \end{array}$$

where S(t) represents the susceptible, U(t) represents the unidentified infected, C(t) represents the confirmed individuals at time *t*, and β and γ are constants; β is the disease transmission rate and γ is the proportion of the confirmed patients out of unidentified infected people. The ratio 1/γ is the average period of time until an unidentified infected person is confirmed. Suppose that the total population *N* is always satisfied, that is, N=S(t)+U(t)+C(t). In the model, the input data are only the total population and the time series data of the COVID-19 confirmed cases. The initial number of the unidentified infected population in an interesting time period is obtained by using the SUC model and the least squares method with the input data.

In this paper, we present a modified SUC model under the assumption that the policy on financial incentives is implemented to control COVID-19 outbreaks. It is assumed that more people who are infected but not identified will be actively tested if financial incentives are provided. Therefore, because it affects γ, the new value of γ, which has changed because of the financial rewards, is called $\gamma_{I}$. We propose an SUC model with incentives

(4) $$\begin{array}{ccc} \frac{dS(t)}{dt} & = & -\beta \frac{S(t)U(t)}{N}, \end{array}$$

(5) $$\begin{array}{ccc} \frac{dU(t)}{dt} & = & \beta \frac{S(t)U(t)}{N}-\gamma_{I}U\left(t\right), \end{array}$$

(6) $$\begin{array}{ccc} \frac{dC(t)}{dt} & = & \gamma_{I}U\left(t\right). \end{array}$$

Note that when $\gamma_{I}\equiv \gamma$, the governing Equations (4)–(6) become the original SUC model [20]. We employ a finite difference method to solve the governing equations. Let Δt be a temporal step size and $S_{n}=S\left(n\Delta t\right)$, $U_{n}=U\left(n\Delta t\right)$, and $C_{n}=C\left(n\Delta t\right)$. The system of Equations (4)–(6) is integrated using the explicit Euler method as follows:

(7) $$\begin{array}{ccc} \frac{S_{n+1}-S_{n}}{\Delta t} & = & -\beta \frac{S_{n}U_{n}}{N}, \end{array}$$

(8) $$\begin{array}{ccc} \frac{U_{n+1}-U_{n}}{\Delta t} & = & \beta \frac{S_{n}U_{n}}{N}-\gamma_{I}U_{n}, \end{array}$$

(9) $$\begin{array}{ccc} \frac{C_{n+1}-C_{n}}{\Delta t} & = & \gamma_{I}U_{n}. \end{array}$$

After rearranging the above equations, we have the following equations:

(10) $$\begin{array}{ccc} S_{n+1} & = & S_{n}-\Delta t\beta \frac{S_{n}U_{n}}{N}, \end{array}$$

(11) $$\begin{array}{ccc} U_{n+1} & = & U_{n}+\Delta t\left(\beta \frac{S_{n}U_{n}}{N}-\gamma_{I}U_{n}\right), \end{array}$$

(12) $$\begin{array}{ccc} C_{n+1} & = & C_{n}+\Delta t\;\gamma_{I}U_{n}. \end{array}$$

We use the following fitting function in MATLAB, lsqcurvefit (MATLAB R2020a), to obtain the optimal parameter values that fit well with the confirmed case data:

(13) $$\begin{array}{c} \left[\beta ,\;\gamma ,\;U_{0}\right]=\mathrm{lsqcurvefit}{(}^{\prime }SUCmodel^{\prime },\;\left[\beta^{0},\;\gamma^{0},\;U_{0}^{0}\right],\;\mathrm{Tdata},\;\mathrm{Cdata},\;\mathrm{lb},\;\mathrm{ub}). \end{array}$$

Here, β,γ, and $U_{0}$ are the optimized parameters. More details about the numerical solution algorithm can be found in [20].

## 3. Computational Experiments

In this paper, the data of the confirmed cases listed in Table A1 are used. The data are only from domestic infections, not from overseas, among total infections in South Korea. We use the following parameters: $N=5\times 10^{7}$, Δt=0.001, $\beta^{0}=1$, $\gamma^{0}=1$, $U_{0}^{0}=0.1C_{0}$, $\mathrm{lb}=(10^{-3},\;10^{-3},\;0.01C_{0})$, and $\mathrm{ub}=(10,\;10,\;5C_{0})$ for all numerical computations.

### 3.1. Estimation without Financial Incentives

**Figure 2:**
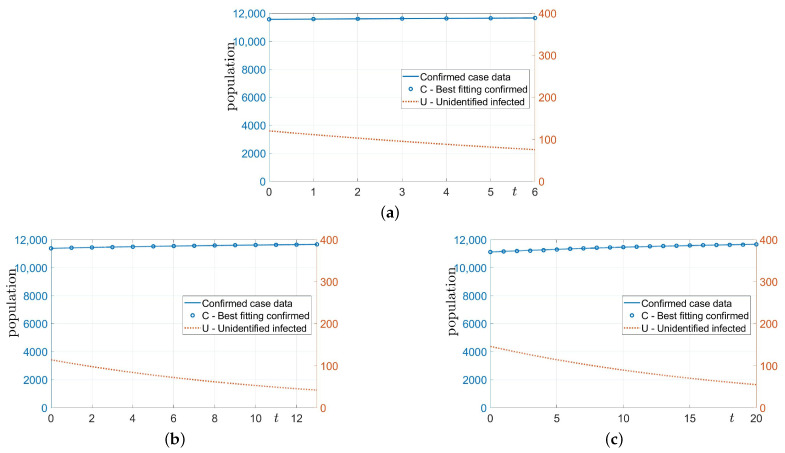
Computational results with (**a**) p=7, (**b**) p=14, and (**c**) p=21.

In this section, using Equations (10)–(13), we estimate the number of unidentified infected populations. Here, $\gamma_{I}=\gamma$ because of the assumption that there are no financial rewards. We consider *p* as the number of Cdata used to evaluate the size of the current unidentified infected population. We use the recent *p* data in Table A1. Figure 2a–c show the computational results with three different *p* values. Here, we take p=7,14, and 21. We observe that the number of confirmed cases increases very slightly, and the number of unconfirmed infected cases decreases over time, as shown in Figure 2. Table 1 lists the computed numbers of the best fitting confirmed and the unidentified infected cases of COVID-19 on 17 July 2020. When the recent small amount of data is used, the results fit well with the time series data.

**Figure 3:**
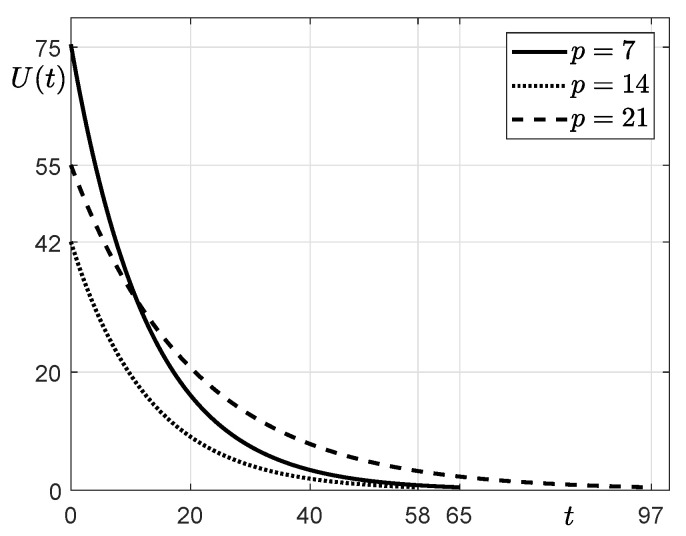
Estimation of the unidentified confirmed patients *U* from 17 July 2020 to the end of the epidemic with different *p* values.

Moreover, using the SUC model with the obtained parameter values (β, γ), we predict the time required to reach the end of the epidemic (called ending time *T* of the spread of COVID-19), which is defined by the days taken for the number of unidentified infected patients to be less than a tolerance from the base date. That is, we calculate the minimum time *T* such that U(T)<tol, where a tolerance, tol, is set to be 0.5. Figure 3 shows the estimation of the unidentified confirmed patients *U* from 17 July 2020 to the end of the epidemic with different *p* values. The times *T* for p=7,14, and 21 are 65,58, and 97, respectively.

### 3.2. Estimation with Financial Incentives

So far, we have estimated the number of unidentified infected cases without financial incentives. Here, we consider the cases where financial incentives are provided. From now on, we assume that if there is a policy that offers financial compensation to the self-reporting people with COVID-19 symptoms and diagnosis of confirmation, then this motivates people for voluntary testing, and they are isolated for treatment. As a result, the number of confirmed patients increases because more people are tested to see if they are infected.

There are two steps to estimate the ending time *T* of the spread of COVID-19.

**Step** **1.** Before the implementation of the incentive policy, we first estimate $\beta ,\;\gamma ,\;S^{n+1},$ and $U^{n+1}$ using the SUC model with the number of confirmed cases up to now. Here, p=7 is fixed.
**Step** **2.** Assuming that financial incentives are provided, we compute S(t),U(t),C(t), and *T* using the SUC model and the iterative method until U(t)<tol. Here, we use the same parameters as those used and obtained in **Step 1** except for γ. Instead of γ, we use $\gamma_{I}$ that is greater than 0.

**Figure 4:**
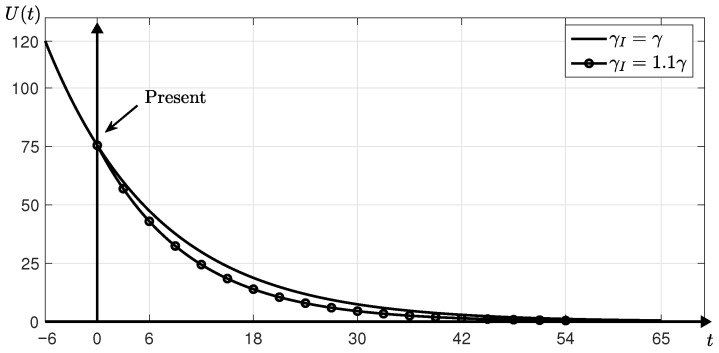
U(t) over time *t* until the epidemic is ended.

We set the initial parameters above at t=0. In **Step 1**, we obtain β≈0.0891 and γ≈0.1665 at t=6. We reset t=6 to t=0. When $\gamma_{I}$ is taken to be the same as γ, the epidemic may be ended at t=65. Assuming that $\gamma_{I}=1.1\gamma \approx 0.1832$ due to an incentive policy, we estimate that the epidemic may end at t=54. Figure 4 shows the numerical results. The increase in $\gamma_{I}$ shortens the average time it takes to identify unconfirmed infected people, which can rapidly isolate the unidentified infected cases. Thus, it prevents the spread of the infectious disease so that it can be seen that the time *T* is reduced.

**Figure 5:**
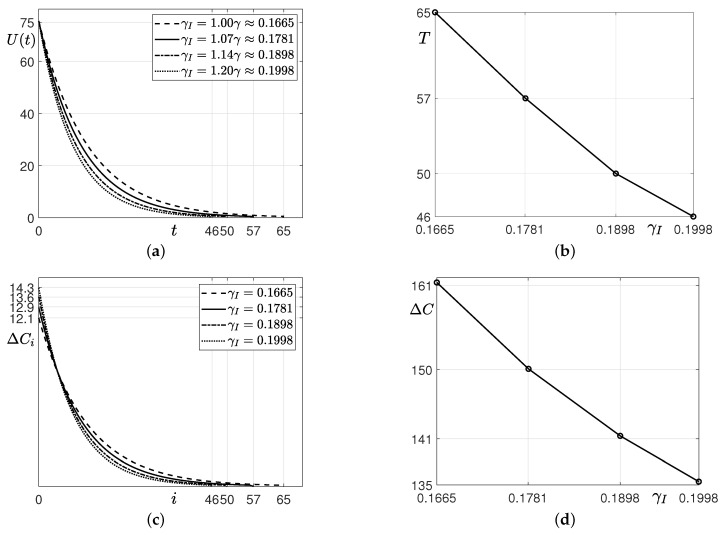
Computational results of (**a**) U(t), (**b**) *T*, (**c**) $\Delta C_{i}$, and (**d**) ΔC with respect to $\gamma_{I}$.

We examine the effect of $\gamma_{I}$, as shown in Figure 5. We observe the estimation for $U,\;T,\;\Delta C_{i},$ and ΔC by changing $\gamma_{I}$ from γ to 1.2γ, i.e., from 0.1665 to 0.1998. Figure 5a shows the changes in the unidentified confirmed patients *U* over time *t* with various $\gamma_{I}$ values. It is observed that *U* decreases rapidly at the beginning as the value of $\gamma_{I}$ increases due to the policy implementation. The larger $\gamma_{I}$, the shorter the time it takes for people in *U* to be confirmed, therefore, they are quickly isolated from their community and *U* decreases faster. As a result, the ending time *T* decreases as γ increases as shown in Figure 5b. In particular, when $\gamma_{I}$ is 1.0, *T* is 65, whereas when $\gamma_{I}$ is 1.07, *T* is greatly reduced to 57. That is, when the value of $\gamma_{I}$ increases by 7% from the current γ, *T* decreases by about 12.3%. Moreover, the larger the value γ, the smaller the decrease in *T*. Figure 5c illustrates the increment of confirmed cases on each day *i* (i=1,2,…,T), which is defined as $\Delta C_{i}=C_{i}-C_{i-1}$. Here, $C_{0}$ is the cumulative number of the confirmed cases when the policy has begun to be applied, and $C_{T}$ is the cumulative number of the confirmed cases up to *T*. Figure 5d shows the increment $\Delta C=C_{T}-C_{0}$ of confirmed cases from a reference time to ending time with respect to $\gamma_{I}$. The increment ΔC decreases as $\gamma_{I}$ increases. Thus, the total number of confirmed cases can be reduced due to the positive effect of the incentive policy.

**Figure 6:**
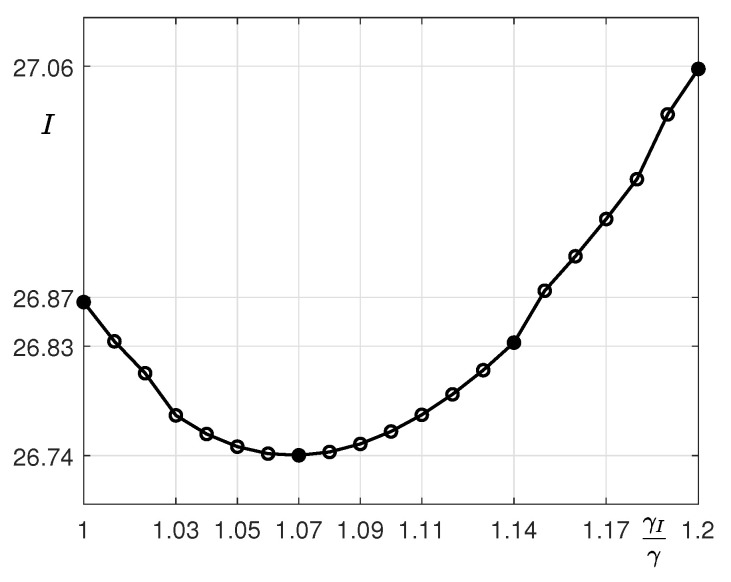
Total incentive *I* with respect to $\gamma_{I}$.

We consider the case in which the total budget for financial incentives is $I=\gamma_{I}\Delta C$ because it is assumed that $\gamma_{I}$ also increases as the total budget *I* decreases slightly and then increases, as shown in Figure 6. When $\gamma_{I}=1.07\gamma$, the total budget *I* is at a minimum, and *I* gradually decreases until it reaches this. When $\gamma_{I}$ is greater than 1.07, *I* increases. Here, the currency is in an arbitrary unit. Even if the total budget of the compensation increases, the epidemic is expected to end sooner. As a result, it is possible to reduce the considerable costs, in terms of human resources and medical equipment, to combat this infectious disease and recover the economic losses associated with it.

## 4. Discussion

We have improved the SUC epidemic model to include the effect of financial incentives, which is one of the efficient methods for controlling COVID-19 outbreaks. We can estimate the number of unidentified people, who are infected with the contagious disease but not confirmed, using the original SUC model. It is important to prevent the highly contagious viral disease spread early in the outbreak by quickly identifying and isolating the confirmed cases, and treating them. Hence, for the early cessation of the epidemic, we consider giving financial incentives to people who have been confirmed with COVID-19 through voluntary testing and have known the route of infection.

In this paper, it is assumed that a policy would be implemented to provide financial rewards to patients who have taken voluntary diagnostic tests for COVID-19 and been confirmed to have the disease. The policy is only valid after its implementation. In addition, it is assumed that there is no moral hazard for rewards by setting specific conditions for compensation and the appropriate compensation amount. As one of the specific conditions of rewards, the incentives are given only if someone knows who the coronavirus was transferred from. Providing adequate compensation can motivate people who would not have revealed their symptoms and have avoided testing, even if an infection is suspected when there is no compensation. Thus, we suppose that the monetary rewards encourage more suspected people to take the tests, leading to more people being confirmed. This is expressed as an increase in the proportion of the confirmed cases out of the unidentified infected cases, that is, γ in the SUC model, due to financial incentives. Then, we investigate the effect of γ on the time *T* it takes for the epidemic to end. We define this as the end of the COVID-19 epidemic if the number of unidentified infected people is less than 1. Using the SUC model, we compute the number of confirmed patients and the time *T* according to the change of the γ value.

The financial incentives are not the only policy aimed at preventing the spread of infectious diseases. For example, an anonymous test can alleviate the worry of being socially criticized. However, by providing financial support to the people for the damage suffered by being isolated due to the confirmed coronavirus, the government can actively encourage people to take voluntary examinations. If this compensation allows early detection of an infected person, there are several major positive effects. Firstly, the damage caused by community transmission can be prevented in advance. Secondly, the considerable costs required to prevent the spread of COVID-19 can be saved. Thirdly, it is possible to reduce the economic loss that can occur in various industries due to the prolonged spread of COVID-19. Lastly, if the unidentified infected people are diagnosed early when symptoms are minimal, this can reduce the fatality rate with the care of medical staff, although there are no treatments for COVID-19 yet. Hence, it is of great significance that we can effectively prevent the spread of the epidemic by using a relatively small amount for financial incentives, compared with the damage caused by the spread of the coronavirus.

This paper has presented the model that can be used to estimate the effect of increasing or decreasing the number of confirmed cases through policy implementation. There are limitations due to the strict constraints on the mathematical model and reliability problems with the collected data; however, it is significant that the model can predict the degree of effectiveness of the policy. In reality, we cannot know in advance the value of $\gamma_{I}$, which varies depending on the amount of incentive. Therefore, in practical situations, it is necessary to first provide compensation, analyze the data of the number of confirmed patients, and then it should be checked whether the amount of compensation is appropriate for controlling the spread of the epidemic to the expected extent. The numerical experiments in this paper can be used as a criterion indicator when implementing policies. Before the first implementation of the financial incentive policy, there is no information on how to set an appropriate incentive amount. The amount of incentive may be determined by considering a factor of wages or minimum cost of living. With at least two policy changes, we can derive an appropriate amount of compensation by using a power cost function.

Let *x* be the financial incentive amount. Here, the currency unit is an arbitrary unit. For instance, as a result of the financial incentive policy implementation, we suppose that $\gamma_{I}$ is $1.1\gamma_{\mathrm{ref}}$ when x=1, and for the higher effect of the policy, we assume $\gamma_{I}=1.2\gamma_{\mathrm{ref}}$ when we raise *x* to 6. Using these data, we can formulate a power cost function $\gamma_{I}$ with respect to *x*:

(14) $$\begin{array}{c} \gamma_{I}\left(x\right)=\gamma_{\mathrm{ref}}\left(1+ax^{b}\right), \end{array}$$

where $\gamma_{\mathrm{ref}}$ is the optimal value γ that was fitted to the confirmed patient data when there is no financial incentive, and *a* and *b* are constants we need to find. By changing the terms and taking log on both sides of Equation (14), we obtain

(15) $$\begin{array}{c} \log\left(\frac{\gamma_{I}\left(x\right)}{\gamma_{\mathrm{ref}}}-1\right)=\log a+b\log x. \end{array}$$

**Figure 7:**
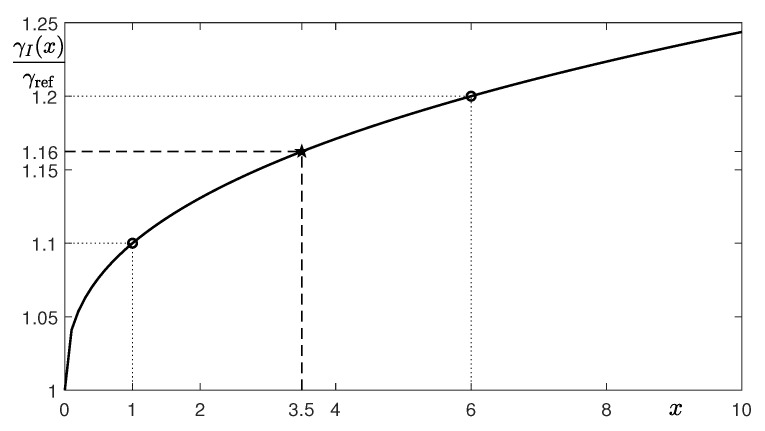
Plot of the fitting function for a power cost function, $\gamma_{I}\left(x\right)/\gamma_{\mathrm{ref}}=1+\exp\left(A\right)x^{b}$.

We define Equation (15) as

$$\begin{array}{c} Y=A+bX, \end{array}$$

where $Y=\log\left(\gamma_{I}\left(x\right)/\gamma_{\mathrm{ref}}-1\right)$, X=logx, and A=loga. The MATLAB function “polyfit” is used to fit the linear polynomial. From our assumptions, $\gamma_{I}\left(1\right)=1.1\gamma_{\mathrm{ref}}$ and $\gamma_{I}\left(6\right)=1.2\gamma_{\mathrm{ref}}$, we get the coefficients A=−2.3026 and b=0.3869. Figure 7 represents the graph of the fitting function $\gamma_{I}\left(x\right)/\gamma_{\mathrm{ref}}=1+\exp\left(A\right)x^{b}$. In this case, for $\gamma_{I}=1.16\gamma_{\mathrm{ref}}$, we can estimate the proper financial incentive as x=3.5.

## 5. Conclusions

In this paper, a modified SUC epidemic model was proposed to control COVID-19 outbreaks by providing financial incentives. The proposed model is based on a recently developed epidemic model, the SUC epidemic model, with which we can estimate the unidentified infected population. It was assumed that financial incentives are provided to confirmed patients after voluntary testing and staff who have made a significant contribution to finding confirmed cases quickly, and the incentives result in a change in the value of γ in the SUC model. The computational results demonstrated that the proposed mathematical epidemic model can effectively control COVID-19 outbreaks through financial incentives. Finally, we discussed how our model can actually be used to establish a financial incentive policy. In the future, the proposed mathematical model can be further modified to include the actual effects of financial incentives on controlling COVID-19 outbreaks.

## Figures and Tables

**Table 1:** Computational results for the best fitting confirmed (*C*) and the unidentified infected (*U*) of COVID-19 with respect to *p*. The confirmed case data were 11,668 on 17 July 2020.

*p*	7	14	21
*C*	11,664	11,664	11,675
*U*	75	42	55

## Data Availability

The data used to support the findings of this study are available from the corresponding author upon request.

## Appendix A. Data

Table A1 lists the data for domestic COVID-19 cases, not including overseas cases, in South Korea reported by KDCA from March 25 to 17 July 2020 [4]. In this paper, the data are used for the numerical simulation.

**Table A1:** Confirmed cases of COVID-19 domestic outbreak in South Korea from 25 March to 17 July 2020 [4].

No	Date	Cases	No	Date	Cases	No	Date	Cases	No	Date	Cases
1	25-March	8910	30	23-April	9677	59	22-May	9941	88	20-June	10,945
2	26-March	8957	31	24-April	9681	60	23-May	9960	89	21-June	10,985
3	27-March	9023	32	25-April	9687	61	24-May	9977	90	22-June	10,996
4	28-March	9115	33	26-April	9688	62	25-May	9990	91	23-June	11,012
5	29-March	9171	34	27-April	9691	63	26-May	10,006	92	24-June	11,043
6	30-March	9185	35	28-April	9693	64	27-May	10,043	93	25-June	11,066
7	31-March	9268	36	29-April	9697	65	28-May	10,111	94	26-June	11,093
8	01-April	9327	37	30-April	9697	66	29-May	10,166	95	27-June	11,124
9	02-April	9375	38	01-May	9698	67	30-May	10,193	96	28-June	11,164
10	03-April	9415	39	02-May	9698	68	31-May	10,208	97	29-June	11,194
11	04-April	9392	40	03-May	9701	69	01-June	10,238	98	30-June	11,217
12	05-April	9433	41	04-May	9701	70	02-June	10,274	99	01-July	11,252
13	06-April	9464	42	05-May	9701	71	03-June	10,320	100	02-July	11,296
14	07-April	9494	43	06-May	9701	72	04-June	10,353	101	03-July	11,348
15	08-April	9523	44	07-May	9702	73	05-June	10,387	102	04-July	11,384
16	09-April	9539	45	08-May	9703	74	06-June	10,430	103	05-July	11,427
17	10-April	9561	46	09-May	9720	75	07-June	10,483	104	06-July	11,449
18	11-April	9579	47	10-May	9746	76	08-June	10,516	105	07-July	11,469
19	12-April	9587	48	11-May	9775	77	09-June	10,551	106	08-July	11,499
20	13-April	9596	49	12-May	9797	78	10-June	10,594	107	09-July	11,526
21	14-April	9611	50	13-May	9819	79	11-June	10,634	108	10-July	11,548
22	15-April	9627	51	14-May	9845	80	12-June	10,677	109	11-July	11,568
23	16-April	9638	52	15-May	9867	81	13-June	10,720	110	12-July	11,589
24	17-April	9646	53	16-May	9876	82	14-June	10,751	111	13-July	11,608
25	18-April	9655	54	17-May	9882	83	15-June	10,774	112	14-July	11,622
26	19-April	9658	55	18-May	9887	84	16-June	10,795	113	15-July	11,633
27	20-April	9664	56	19-May	9896	85	17-June	10,826	114	16-July	11,647
28	21-April	9668	57	20-May	9920	86	18-June	10,877	115	17-July	11,668
29	22-April	9673	58	21-May	9930	87	19-June	10,909			

## Appendix B. MATLAB Code

The MATLAB code used in this paper is available from the website of the corresponding author:

http://elie.korea.ac.kr/~cfdkim/codes/

The main code is as follows:

clear; format long; global N C0; Cs=[8910 8957 9023 9115 9171 9185 9268 9327 9375 9415 9392 9433 …    9464 …  9494 9523 9539 9561 9579 9587 9596 9611 9627 9638 9646 9655 9658 …      …  9664 9668 9673 9677 9681 9687 9688 9691 9693 9697 9697 9698 9698 …      …  9701 9701 9701 9701 9702 9703 9720 9746 9775 9797 9819 9845 9867 …      …  9876 9882 9887 9896 9920 9930 9941 9960 9977 9990 10006 10043 …  10111 10166 10193 10208 10238 10274 10320 10353 10387 10430 …    10483 …  10516 10551 10594 10634 10677 10720 10751 10774 10795 10826 …    10877 …  10909 10945 10985 10996 11012 11043 11066 11093 11124 11164 …    11194 …  11217 11252 11296 11348 11384 11427 11449 11469 11499 11526 …    11548 …  11568 11589 11608 11622 11633 11647 11668]; ts=[1:length(Cs)]; given=ts(end); p=7; N=5.0e+7; Tdata=ts(given+1-p:given); Tdata=Tdata-Tdata(1); Cdata=Cs(given+1-p:given); pp=Tdata(end); C(1)=Cdata(1); C0=C(1); b0=1.0; g0=1.0; Us=0.1*C0; param=lsqcurvefit(@SUCmodel,[b0 g0 Us],Tdata,Cdata,…  [1.0e-3 1.0e-3 0.01*C0],[10 10 5*C0]); b=param(1); g=param(2); U(1)=param(3); S(1)=N-U(1)-C(1); Nt=1000*pp; dt=0.001; for i=1:Nt  S(i+1)=S(i)-dt*b*S(i)*U(i)/N;  U(i+1)=U(i)+dt*(b*S(i)*U(i)/N-g*U(i));
end
C=N-S-U; figure(1); clf; grid on; box on; hold on yyaxis left; plot(Tdata,Cdata,’ko’,’linewidth’,2); t=linspace(0,p,Nt+1); plot(t,C,’k’,’linewidth’,1.5); axis([t(1) t(end) 0 1.1*C(end)]); xlabel(’t’); ylabel(’population’) yyaxis right; plot(t,U,’k:’,’linewidth’,2); axis([t(1) t(end) 0 0.1*C(end)]); legend(’Confirmed case data’,’C - Best fitting confirmed’, …  ’U - Unidentified infected’,’Location’,’east’)
% Given gamma after the implementation of incentive policy, find U
G=[1:0.1:1.2]*g; iter=length(G); Nt=1000; dt=0.001; tol=0.1; figure(2); clf; grid on; box on; hold on; xlabel(’t’); ylabel(’UU’) for k = 1:iter  tt=[]; SS=[]; UU=[]; CC=[];  j=1; tt(1)=0; SS(1)=S(end); UU(1)=U(end);  while UU(j)>=tol    for i = j:j+Nt-1      SS(i+1)=SS(i)-dt*b*SS(i)*UU(i)/N;      UU(i+1)=UU(i)+dt*(b*SS(i)*UU(i)/N-G(k)*UU(i));      tt(i+1)=tt(i)+dt;    end    j=j+Nt;  end  CC=N-SS-UU; T(k)=round(tt(end)); DC(k)=CC(end)-CC(1);  figure(2); plot(tt,UU,’linewidth’,1.5); hold on
end

% function part
function f = SUCmodel(Parameter, Tdata) global N C0; b=Parameter(1); g=Parameter(2); U(1)=Parameter(3); C(1)=C0; S(1)=N-C(1)-U(1); Nt=1000*Tdata(end); dt=0.001; for i=1:Nt  S(i+1)=S(i)-dt*b*S(i)*U(i)/N;  U(i+1)=U(i)+dt*(b*S(i)*U(i)/N-g*U(i));
end
C=N-S-U; t=linspace(0,Tdata(end),Nt+1); f=interp1(t,C,Tdata);
end

## References

[1] Lai C.C., Shih T.P., Ko W.C., Tang H.J., Hsueh P.R. (2020). Severe acute respiratory syndrome coronavirus 2 (SARS-CoV-2) and corona virus disease-2019 (COVID-19): The epidemic and the challenges. Int. J. Antimicrob. Agents.

[2] Younes A.B., Hasan Z. (2020). COVID-19: Modeling, prediction, and control. Appl. Sci..

[3] World Health Organization (WHO) Coronavirus Disease 2019 (COVID-19) Situation Report 179, accesed 17 July 2020. https://www.who.int/docs/default-source/coronaviruse/situation-reports/20200717-covid-19-sitrep-179.pdf?sfvrsn=2f1599fa_28.

[4] Novel Coronavirus (COVID-19) Situation Reports Published by the Korea Disease Control and Prevention Agency (KDCA). http://www.kdca.go.kr/board/board.es?mid=a20501000000&bid=0015.

[5] Khalifa S.A., Mohamed B.S., Elashal M.H., Du M., Guo Z., Zhao C., Musharraf S.G., Boskabady M.H., El-Seedi H.H.R., Efferth T. (2020). Comprehensive overview on multiple strategies fighting COVID-19. Int. J. Environ. Res. Public Health.

[6] Ha B.T.T., La Quang N., Mirzoev T., Tai N.T., Thai P.Q., Dinh P.C. (2020). Combating the COVID-19 Epidemic: Experiences from Vietnam. Int. J. Environ. Res. Public Health.

[7] Pung R., Chiew C.J., Young B.E., Chin S., Chen M.I., Clapham H.E., Cook A.R., Maurer-Stroh S., Toh M.P.H.S., Poh C. (2020). Investigation of three clusters of COVID-19 in Singapore: Implications for surveillance and response measures. Lancet.

[8] Chu D.K., Akl E.A., Duda S., Solo K., Yaacoub S., Schünemann H.J. (2020). Physical distancing, face masks, and eye protection to prevent person-to-person transmission of SARS-CoV-2 and COVID-19: A systematic review and meta-analysis. Lancet.

[9] Guzzetta G., Poletti P., Ajelli M., Trentini F., Marziano V., Cereda D., Tirani M., Diurno G., Bodina A., Barone A. (2020). Potential short-term outcome of an uncontrolled COVID-19 epidemic in Lombardy, Italy, February to March 2020. Eurosurveillance.

[10] Xie K., Liang B., Dulebenets M.A., Mei Y. (2020). The impact of risk perception on social distancing during the COVID-19 pandemic in China. Int. J. Environ. Res. Public Health.

[11] Ghinai I., McPherson T.D., Hunter J.C., Kirking H.L., Christiansen D., Joshi K., Rubin R., Morales-Estrada S., Black S.R., Pacilli M. (2020). First known person-to-person transmission of severe acute respiratory syndrome coronavirus 2 (SARS-CoV-2) in the USA. Lancet.

[12] Peeri N.C., Shrestha N., Rahman M.S., Zaki R., Tan Z., Bibi S., Baghbanzadeh M., Aghamohammadi N., Zhang W., Haque U. (2020). The SARS, MERS and novel coronavirus (COVID-19) epidemics, the newest and biggest global health threats: What lessons have we learned?. Int. J. Epidemiol..

[13] Day M. (2020). Covid-19: Identifying and isolating asymptomatic people helped eliminate virus in Italian village. BMJ.

[14] Sjödin H., Wilder-Smith A., Osman S., Farooq Z., Rocklöv J. (2020). Only strict quarantine measures can curb the coronavirus disease (COVID-19) outbreak in Italy, 2020. Eurosurveillance.

[15] Han D., Shao Q., Li D., Sun M. (2020). How the individuals’ risk aversion affect the epidemic spreading. Appl. Math. Comput..

[16] Reuters News. https://www.reuters.com/article/us-china-health-rewards/china-city-offers-1400-reward-for-virus-patients-who-report-to-authorities-idUSKCN20L0GE.

[17] Chamie G., Ndyabakira A., Marson K.G., Emperador D.M., Kamya M.R., Havlir D.V., Kwarisiima D., Thirumurthy H. (2020). A pilot randomized trial of incentive strategies to promote HIV retesting in rural Uganda. PLoS ONE.

[18] Adamson B., El-Sadr W., Dimitrov D., Gamble T., Beauchamp G., Carlson J.J., Garrison L., Donnell D. (2019). The cost-effectiveness of financial incentives for viral suppression: HPTN 065 study. Value Health.

[19] Rat-Aspert O., Fourichon C. (2010). Modelling collective effectiveness of voluntary vaccination with and without incentives. Prev. Vet. Med..

[20] Lee C., Li Y., Kim J. (2020). The susceptible-unidentified infected-confirmed (SUC) epidemic model for estimating unidentified infected population for COVID-19. Chaos Soliton. Fract..

[21] Capasso V., Serio G. (1978). A generalization of the Kermack–McKendrick deterministic epidemic model. Math. Biosci..

[22] Gray A., Greenhalgh D., Hu L., Mao X., Pan J. (2011). A stochastic differential equation SIS epidemic model. SIAM J. Appl. Math..

[23] Ma Y., Liu J.B., Li H. (2018). Global dynamics of an SIQR model with vaccination and elimination hybrid strategies. Mathematics.

[24] Khan M.A., Khan Y., Islam S. (2018). Complex dynamics of an SEIR epidemic model with saturated incidence rate and treatment. Physica A.

[25] Wu M., Dai W., Lu Z., Zhao Y., Wang M. (2019). The method for risk evaluation in assembly process based on the discrete-time SIRS epidemic model and information entropy. Entropy.

[26] Nistal R., De la Sen M., Alonso-Quesada S., Ibeas A. (2019). On a new discrete SEIADR model with mixed controls: Study of its properties. Mathematics.

